# Genomic Selection Using Pedigree and Marker-by-Environment Interaction for Barley Seed Quality Traits From Two Commercial Breeding Programs

**DOI:** 10.3389/fpls.2020.00539

**Published:** 2020-05-08

**Authors:** Theresa Ankamah-Yeboah, Lucas Lodewijk Janss, Jens Due Jensen, Rasmus Lund Hjortshøj, Søren Kjærsgaard Rasmussen

**Affiliations:** ^1^Department of Plant and Environmental Sciences, Faculty of Science, University of Copenhagen, Frederiksberg, Denmark; ^2^Center for Quantitative Genetics and Genomics, Department of Molecular Biology and Genetics, Aarhus University, Aarhus, Denmark; ^3^Nordic Seed A/S, Odder, Denmark; ^4^Sejet Plant Breeding, Horsens, Denmark

**Keywords:** genomic selection, prediction ability, barley, pedigree, marker, genotype-by-environment, seed quality traits, *Hordeum vulgare*

## Abstract

With the current advances in the development of low-cost high-density array-based DNA marker technologies, cereal breeding programs are increasingly relying on genomic selection as a tool to accelerate the rate of genetic gain in seed quality traits. Different sources of genetic information are being explored, with the most prevalent being combined additive information from marker and pedigree-based data, and their interaction with the environment. In this, there has been mixed evidence on the performance of use of these data. This study undertook an extensive analysis of 907 elite winter barley (*Hordeum vulgare* L.) lines across multiple environments from two breeding companies. Six genomic prediction models were evaluated to demonstrate the effect of using pedigree and marker information individually and in combination, as well their interactions with the environment. Each model was evaluated using three cross-validation schemes that allows the prediction of newly developed lines (lines that have not been evaluated in any environment), prediction of new or unobserved years, and prediction of newly developed lines in unobserved years. The results showed that the best prediction model depends on the cross-validation scheme employed. In predicting newly developed lines in known environments, marker information had no advantage to pedigree information. Predictions in this scenario also benefited from including genotype-by-environment interaction in the models. However, when predicting lines and years not observed previously, marker information was superior to pedigree data. Nonetheless, such scenarios did not benefit from the addition of genotype-by-environment interaction. A combination of pedigree-based and marker-based information produced a similar or only marginal improvement in prediction ability. It was also discovered that combining populations from the different breeding programs to increase training population size had no advantage in prediction.

## Introduction

In the last two decades, plant breeding programs have advanced from conventional approaches involving visual selection and trait screening over several generations to potentially faster methods by means of marker technologies (Crossa et al., 2017). Genomic selection, the concept of using dense molecular genome-wide data to predict the performance of individuals in a breeding population (Meuwissen et al., 2001), has become more popular in cereal breeding in recent years. Empirical studies in barley (Zhong et al., 2009; Sallam et al., 2015; Nielsen et al., 2016; Thorwarth et al., 2017), wheat (*Triticum aestivum*; Crossa et al., 2010; Dong et al., 2018; Haile et al., 2018; Norman et al., 2018), maize (*Zea mays*; Zhao et al., 2012; Shikha et al., 2017; Vélez-Torres et al., 2018), rice (*Oryza sativa*; Spindel et al., 2015; Xu et al., 2018), and sorghum (*Sorghum bicolor*; De Oliveira et al., 2018; Hunt et al., 2018) have recently all proven that with current advances in the development of high-density array-based DNA marker technologies and reduced costs, genomic selection has become an important tool in cereal breeding.

Prediction models that use realized relationships based on marker information lead to a substantial increase in the prediction accuracies of complex traits compared to those using relationships based on pedigree information (VanRaden et al., 2009), and this has been observed in several genomic selection studies (Crossa et al., 2010; Albrecht et al., 2011; Burgueño et al., 2012). It is assumed that by using the marker-based related matrix, it is possible to account for Mendelian segregation of alleles along with full-sibs, which are more or less expected due to random chance. Hence, while pedigree-based breeding values of unphenotyped lines will only reflect mid-parent genetic contributions, genomic-estimated breeding values of unphenotyped full sibs will reflect genetic differences caused by Mendelian sampling (Velazco et al., 2019).

Other researchers have found less conclusive results, which show that in some instances, pedigree-based relationships may perform similarly to or even better than marker-based relationships in terms of prediction accuracy (Juliana et al., 2017; Hunt et al., 2018). This is particularly true when the pedigree data are very precise and include several generations (Juliana et al., 2017). Numerous studies have also reported the benefits of jointly using marker and pedigree-based relationships in prediction models (Crossa et al., 2010; Albrecht et al., 2011; Burgueño et al., 2012; Rodríguez-Ramilo et al., 2014; Sukumaran et al., 2017). The inclusion of both marker and pedigree has the potential to increase prediction performance because the pedigree information can account for variations that may not be captured by markers at population and family level. Hence they provide better estimates of genetic variations, thereby improving predictive performance (Velazco et al., 2019). However, it has also been demonstrated that when high-quality genome-wide marker and pedigree data are available, it is not always expected that the combination of marker and pedigree information will outperform the use of marker information alone in mixed models due to information matrix redundancy (Albrecht et al., 2011).

In the past, genomic prediction models have been developed to suit single environment predictions. However, in most plant breeding programs, multi-environment trials play a crucial role in the assessment of lines to determine the performance of stability across environments of the prospect variety. The effects of genes on traits are usually influenced by environmental conditions, leading to high levels of genotype-by-environment interactions (Burgueño et al., 2012). Genomic selection models have therefore been extended to fit multi-environment settings. The incorporation of genotype-by-environment interactions (marker-by-environment; GxE and pedigree-by-environment; AxE) in prediction models has been found to increase the performance of such models in comparison with single environment models (Burgueño et al., 2012). Furthermore, it has been shown that prediction accuracy in barley increases when considering marker and marker-by-environment interactions using ridge-regression best linear unbiased prediction to extend genomic selection to multiple environments (Oakey et al., 2016). However, when high dimensional genetic and environmental variables are involved, modeling all possible interactions becomes a challenge. Other studies (Jarquín et al., 2014; Pérez-Rodríguez et al., 2015) have also shown that incorporating interactions between genetic information and environment increased prediction accuracy by a considerable margin. In similar studies, interactions between genetic information based on both pedigree and marker and on 18 environment variables increased the prediction ability (Sukumaran et al., 2017).

The present study assessed the performance of prediction models on four seed quality traits in winter barley from two breeding companies in Denmark: Nordic Seed (hereafter referred to as NS) and Sejet Plant Breeding (hereafter referred to as SJ). Specifically, this study: (i) evaluated the individual and combined effect of marker and pedigree data on prediction ability under different cross-validation schemes, (ii) determined genetic (marker and pedigree) and environment-interaction effects on prediction ability under different cross validation schemes, and (iii) assessed the advantage of increasing training set size by combining lines from different breeding programs.

## Materials and Methods

### Phenotypic Data

Data were provided by two commercial breeding companies in Denmark: NS and SJ (Supplementary Material Table 1: “Phenotypic data”). Line records (1,850 in all) were obtained from 484 commercial elite two-row winter barley lines from NS and evaluated over 3 years (2015, 2016, and 2017) across three locations in Denmark. All three locations were the same for the 3 years. Data from SJ consisted of 964 records from 428 lines and phenotyped for protein content and test weight. Seed fraction weight records for SJ were measured in the Department of Plant and Environmental Sciences at University of Copenhagen, Denmark. The 428 lines from SJ were also grown in 3 years (2015, 2016, and 2017) across two locations in Denmark (except in 2016, where lines were evaluated at only one location).

At each location in the breeding companies, lines were grown in individual experiments consisting of 5 to 20 small trials with the number of lines per each trial ranging between 25 to 30. Two to 6 lines were used as checks in each of the experiment. Each trial was a randomized block-design with three replicates per each line. Management of experiments were the same for all locations within the specific breeding companies. Due to this, there is the possibility of differences in the management practices observed in the different breeding programs.

Five lines were common between the two companies. In all, 2,814 records from 907 lines were used in the analysis. On average, there were 14 distinct environments (company-year-locations). The number of observations recorded in each environment is presented in Table 1. Each line record was phenotyped for protein content (% dry matter), test weight (kg/hL), percentage weight of seed fractions above 2.5 mm in diameter (hereafter referred to as SF_abv2.5), and seed fractions above 2.2 mm in diameter (hereafter referred to as SF_abv2.2). Protein content and test weight were measured using a FOSS Grain Analyzer instrument based on near-infrared transmission technology. The seed fractions were obtained using a SORTIMAT screening instrument to separate seed samples of approximately 100 *g* into four seed size classes of >2.8, 2.8–2.5, 2.5–2.2, and <2.2 mm in diameter. The seed size classes were divided into two main classes: those above 2.5 mm in diameter (>2.8 and 2.8–2.5 mm classes) and those above 2.2 mm in diameter (>2.8, 2.8–2.5, and 2.5–2.2 classes). Their percentage weights were calculated and used in the analysis.

**TABLE 1:** Description of environments and number of observations in each environment.

Company	Year	Location	Environments	Number of observations per environment			
				
				Protein	Test weight	SF_abv2.5	SF_abv2.2
NS	2015	1	NS15_1	191	190	191	191
NS	2015	2	NS15_2	192	192	192	192
NS	2015	3	NS15_3	192	190	192	192
NS	2016	1	NS16_1	240	239	240	240
NS	2016	2	NS16_2	239	222	240	240
NS	2016	3	NS16_3	236	149	240	240
NS	2017	1	NS17_1	185	166	185	185
NS	2017	2	NS17_2	185	133	185	185
NS	2017	3	NS17_3	185	168	185	185
SJ	2015	1	SJ15_1	218	218	230	230
SJ	2015	2	SJ15_2	231	231	231	231
SJ	2016	1	SJ16_1	215	215	215	215
SJ	2017	1	SJ17_1	144	144	144	144
SJ	2017	2	SJ17_2	144	144	144	144

### Genotype information

Lines from both companies were genotyped with the 9K iSelect single-nucleotide polymorphism (SNP) chip by TraitGenetics. Filtering out monomorphic sites and more than 10% missing markers reduced the SNP markers to 4,830 for analysis (Supplementary Material Table 1: “Genotypic data”). The remaining missing SNPs were imputed with “synbreed” using the “random” algorithm, where missing values for a marker were sampled from the marginal allele distribution of that marker (Wimmer et al., 2012). The realized genomic relationship matrix based on marker information was computed (VanRaden, 2008).

### Pedigree Information

Pedigree information on all the lines was provided for the analysis. Pedigree information of the 484 lines in the NS data extended to five generations. It comprised 80 subfamilies in total, with progenies per subfamily (full siblings) ranging between 2 and 24, and 19 lines representing individual crosses (Supplementary Material Table 1: “Pedigree matrix_NS data”). The pedigree information for all 428 lines from SJ extending up to seven generations comprised 106 subfamilies in total (Supplementary Material Table 1: “Pedigree matrix_SJ data”). The number of progenies per subfamily ranged from 2 to 13 full siblings, with 67 lines with individual crosses (no siblings). This shows that although the number of subfamilies within NS was small compared to SJ, the subfamily units within NS had more siblings than SJ.

### Statistical Models

Data were analyzed using mixed linear models, where the main effect of lines, environments, markers, pedigree, and their interactions with environments were modeled using random covariance structures. Since data on line records, marker and pedigree information were used, the line was included as main effect in all models so as to partition the variance components into additive (marker and pedigree), and non-additive (residual genetic) effects. The seed quality traits analyzed were protein content, test weight and two seed fraction weights. The models used for evaluation are briefly described below.

Model 1: ELG

In this model, a linear mixed model that accounts for main environmental effects, line effects, and marker effects was fitted as expressed below:

(1) $$y_{i\,j}=\mu +E_{i}+L_{j}+g_{j}+\varepsilon_{i\,j}$$

where *y*_*ij*_, is the phenotype, μ is the intercept, *E*_*i*_ is the random effect of the *i*th environment (company-year-location), *L*_*j*_ is the random effect of the *j*th line, and ε_*ij*_ is the residual error. *g*_*j*_ is the random “genomic breeding value” of the *j*th line. This accounts for the marker-based relationships of the lines by following a multivariate normal density, with zero mean and covariance matrix $\text{Cov}\,\left(g\right)=G\,\sigma_{g}^{2}$, where *G* is the realized genomic relationship matrix derived by the VanRaden formula $\frac{X\,X^{\prime }}{2\,\sum p_{m}\,\left(1-p_{m}\right)}$ (VanRaden, 2008), where *X* is the centered and standardized marker matrix, *p*_*m*_ is the allele frequency of the *m*th marker, and $\sigma_{g}^{2}$ is the marker genetic variance. All other random effects in the above model follow normal independent and identical distributions, such that $E_{i}∼N\,\left(0,I\,\sigma_{E}^{2}\right)$, $L_{j}∼N\,\left(0,I\,\sigma_{L}^{2}\right)$, and $\varepsilon_{i\,j}∼N\,\left(0,I\,\sigma_{\varepsilon }^{2}\right)$.

Model 2: ELG-GxE

This model was obtained by extending model 1 by incorporating the interaction between the random effects of the markers and the environments, such that the model accounts for the main effects of the environments, the main effects of the lines, the main effects of the markers, and the interactions between the markers and the environments. The interaction term is incorporated as a Hadamard product of two covariance structures (*E*_*i*_and*g*_*j*_) describing relationships between the environments and the lines based on the marker information. Model 2 is expressed as:

(2) $$y_{i\,j}=\mu +E_{i}+L_{j}+g_{j}+g\,E_{i\,j}+\varepsilon_{i\,j}$$

where *gE*_*ij*_ is the interaction between the *j*th line based on the marker data in the *i*th environment. The other components of the model are as defined previously. The random vector containing the interaction term is assumed to follow a multivariate normal distribution gEi⁢j∼N(0,[ZgGZg′][ZEZE′]∘σg⁢E2), where *Z*_*g*_ is the incidence matrix for the vector of marker effects, *Z*_*E*_ is the incidence matrix of the environmental effects, $\sigma_{g\,E}^{2}$ is the variance of the interaction term *gE*_*ij*_, and ° represents the cell-by-cell or Hadamard product between the two matrices.

Model 3: ELA

Model 3 was obtained by replacing the random effect of the marker covariate in model 1 with the random additive relationship of the lines based on the pedigree information (*a*). Model 3 is represented as:

(3) $$y_{i\,j}=\mu +E_{i}+L_{j}+a_{j}+\varepsilon_{i\,j}$$

where *a*_*j*_ is the random additive effect of the *j*th line, with a covariance structure based on the pedigree relationships between the lines, so that *a*_*j*_ follows a multivariate normal density with zero mean and covariance matrix $\text{Cov}\,\left(a\right)=A\,\sigma_{a}^{2}$, where *A* is the additive pedigree-relationship matrix, and $\sigma_{a}^{2}$ is the pedigree genetic variance.

Model 4: ELA-AxE

Model 4 was obtained by adding the interaction between the lines based on their pedigree information (*a*) and the environments (*E*) to model 3. As in model 2, the interaction was incorporated as the Hadamard product of the covariance structures for *E*_*i*_and*a*_*j*_ and is thereby represented as:

(4) $$y_{i\,j}=\mu +E_{i}+L_{j}+a_{j}+a\,E_{i\,j}+\varepsilon_{i\,j}$$

where *aE*_*ij*_ is the interaction term between the *j*th line based on the pedigree information in the *i*th environment assumed to follow a multivariate normal distribution of the form aEi⁢j∼N(0,[ZaAZa′][ZEZE′]∘σa⁢E2). *Z*_*a*_ is the incidence matrix for the vector of pedigree effects, and $\sigma_{a\,E}^{2}$ is the variance of the interaction term *aE*_*ij*_.

Model 5: ELGA

This model was obtained by combining the components of model 1 and model 3, such that the model accounts for random effects of the environments (*E*), lines (*L*), marker (*g*), and pedigree (*a*). Model 5 is represented as:

(5) $$y_{i\,j}=\mu +E_{i}+L_{j}+g_{j}+a_{j}+\varepsilon_{i\,j}$$

where all the terms of this model are as previously described.

Model 6: ELGA-GxE-AxE

Model 6 is literally the combination of the components in models 1 to 5, making it the most comprehensive model. With all the terms previously defined, the model is expressed as.

(6) $$y_{i\,j}=\mu +E_{i}+L_{j}+g_{j}+a_{j}+g\,E_{i\,j}+a\,E_{i\,j}+\varepsilon_{i\,j}$$

All the above models were fitted in *R* using the Bayesian Generalized Linear Regression (BGLR) package (Pérez and de los Campos, 2014; de los Campos and Pérez-Rodríguez, 2018).

### Assessing Prediction Performance for Different Cross-Validation Strategies

Genomic prediction models were initially fitted with data from each company and a combined dataset to estimate the variance components for each model. To assess the performance of all the models for each breeding program separately, three cross-validation schemes were implemented.

(i) CV1: Random 10-fold cross validation. This scheme mimics breeding problem of predicting the performance of newly developed lines that have not yet been observed in any environment (Jarquín et al., 2017). Here, lines were randomly partitioned into 10 folds and nine folds (90% of the lines) were used to train the model to predict the remaining one fold (10% of the lines). The partitioning was done such that all line records from the same genotype appear in the same fold. The process was repeated ten times until each fold had been used once as the validation set. The correlation between the observed phenotypic measures and the estimated breeding values ($\hat{k}$) for each genotype (*r*_*y,$\hat{k}$*_) was estimated and the average of the correlation coefficients from the ten folds reported as the prediction ability. The estimated breeding value $\hat{k}$ is defined in respect to the specific model used. Standard error from the mean of the 10-folds correlations were recorded.
(ii) CV0: Predicting unobserved or new year. This scheme allows the prediction of crop performance of all lines in unobserved (future) year. Also referred to as forward prediction or leave-one-year-out, this scheme allows the prediction of environments in a given year using all the environments from the previous years. In this study, data from 2015 and 2016 were combined to form the training set, and year 2017 was used as the validation set. For prediction with SJ data, there were 303 and 125 in the training and validation sets, respectively, while for NS, there were 317 and 167 lines in the training and validation sets, respectively. This scheme provides a more realistic problem often faced by breeders which has to do with unobserved years. The success of this strategy depends not only on the number of related lines in the training and validation sets but mostly on the correlation between the environmental conditions of the unobserved years and the previous years (Jarquín et al., 2017).
(iii) CV00: Predicting newly developed lines in new year. This scheme aims at predicting the performance of all new lines in the future year. This scheme is similar to CV0, however, the lines to be predicted have never been observed in the years previously. This scheme applies to breeding situations where most of the materials have not been observed in previous fields but their performance needs to be estimated for the next year (Jarquín et al., 2017; Howard et al., 2019). Here lines that were evaluated only in 2017 but not in any of the previous years were used as validation set while the rest were used as the training set. For SJ predictions, there were 348 and 80 lines in the training and validation sets, respectively, while for NS there were 374 and 110 lines in the training and validation sets, respectively.

In CV0 and CV00, the procedure did not involve random partitioning, and hence implemented once. The prediction ability was estimated as correlation between the predicted values and the observed phenotypic values. Predictions using individual breeding program data were referred to as within-company predictions.

Subsequently, we assessed the effect of combining different populations originating from different programs to increase training population size in prediction. We referred to these scenarios as across-company predictions while those using data specific to the different breeding programs are referred to as within-company predictions. In the across-company predictions, data from both breeding programs were combined and lines were randomly divided into ten folds. Folds were created such that nine of them contain lines from both companies, while the remaining fold contain either (i) only lines from SJ as the validation set or (ii) only lines from NS as the validation set. All models previously discussed were modified by including breeding program as a fixed effect in the across-company predictions. Predictions derived from this approach were compared to the ones derived in the CV1 of within-company predictions discussed above.

### Estimating Heritability From Unbalanced Data

Broad-sense (*H*^2^) and narrow-sense (*h*^2^) heritabilities were estimated, respectively, as:

(7) $$H^{2}=\frac{\sigma_{L}^{2}+\sigma_{g}^{2}+\sigma_{a}^{2}}{\sigma_{y}^{2}}\,\text{and}\,h^{2}=\frac{\sigma_{g}^{2}+\sigma_{a}^{2}}{\sigma_{y}^{2}}$$

where phenotypic variance was computed as, $\sigma_{y}^{2}=\sigma_{g}^{2}+\sigma_{a}^{2}+\left(\sigma_{g\,E}^{2}/m_{h}\right)+\left(\sigma_{a\,E}^{2}/m_{h}\right)+\left(\sigma_{R}^{2}/p_{h}\right)$. For models containing marker relationships alone, the value $\sigma_{a}^{2}=0$ was used, and for models containing pedigree relationships alone, the value $\sigma_{g}^{2}=0$ was used. Furthermore, the values $\sigma_{g\,E}^{2}=0$ and $\sigma_{a\,E}^{2}=0$ were used for models without environmental interactions. Given that the analysis was based on unbalanced data, the equation was followed with the definition mh=n∑i=1n1mi and ph=n∑i=1n1pi where *n* is the number of genotypes, *m*_*i*_ is the number of environments for each genotype, and *p*_*i*_ is the number of plots across environments for each genotype (Holland et al., 2003).

## Results

### Descriptive Statistics

The lines used in this study came from active barley breeding programs run by two breeding companies. In each breeding company, lines were grown between one and three locations over three years, comprising a total of 14 environments (company-year-location combinations), with NS having nine environments and SJ five environments. Table 2 gives the summary description of the respective traits. Overall, the average protein content was 10.48% (SD = 0.83), and that of NS and SJ was 10.54% (SD = 0.69) and 10.36% (SD = 1.04), respectively. The average and standard deviation estimates of the remaining traits given in the table shows that seed fraction SF_abv2.5 was the most variable, followed by protein, and with the least variable being seed fraction SF_abv2.2.

**TABLE 2:** Summary description of traits for overall data and by breeding company.

	Mean	SD	Min.	Max.
**Combined**				
Protein (%)	10.48	0.83	8.58	13.40
Test weight (kg/hL)	64.33	4.10	51.60	75.59
SF_abv2.5 (%)	83.36	14.59	23.77	99.70
SF_abv2.2 (%)	97.03	3.18	77.64	99.98
**NS**				
Protein (%)	10.54	0.69	8.70	13.40
Test weight (kg/hL)	62.84	3.21	51.60	70.50
SF_abv2.5 (%)	82.88	14.86	23.77	99.70
SF_abv2.2 (%)	96.92	3.17	77.64	99.98
**SJ**				
Protein (%)	10.36	1.04	8.58	13.33
Test weight (kg/hL)	66.89	4.22	55.75	75.59
SF_abv2.5 (%)	84.30	14.04	37.20	99.20
SF_abv2.2 (%)	97.24	3.20	79.82	99.87

**FIGURE 1:**
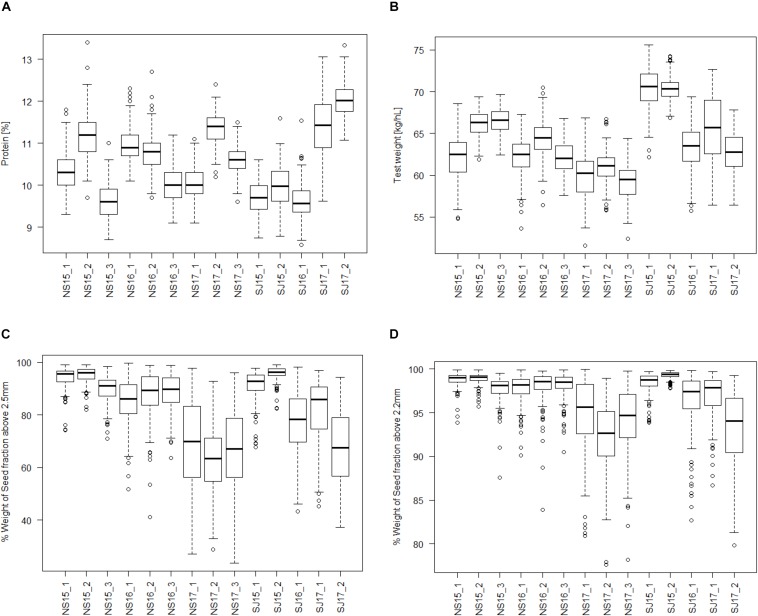
Boxplot of seed quality trait distributions among the 14 environments (company-year-location). **(A)** Protein content, **(B)** test weight, **(C)** seed fraction SF_abv2.5; and **(D)** seed fraction SF_abv2.2.

Figure 1 presents a boxplot of all the traits analyzed for each of the 14 environments in the study. Except for protein content, all the traits showed higher values in 2015 than in 2017 for both breeding companies. For protein and test weight, the variations in the environments seem dependent on both year and location, while for the seed fractions it seems more dependent on year in both breeding companies. The boxplot shows significant environmental effects in all the traits. Analysis of variance of the 14 environments shows F values of 510, 443, 260, and 163 for protein, test weight, SF_abv2.5, and SF_abv2.2, respectively.

### Relationship Between Lines

**FIGURE 2:**
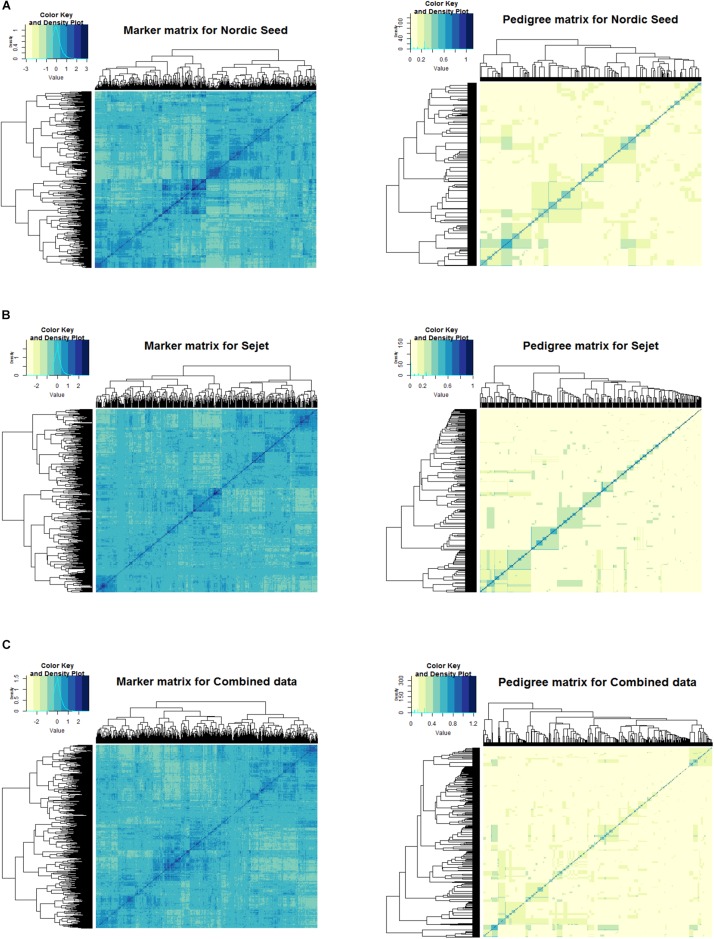
Heat map of marker (left) and pedigree (right) relationship matrices for Nordic Seed (NS), Sejet (SJ), and combined data. **(A)** NS data, **(B)** SJ data, and **(C)** combined data.

The degree of relatedness of the lines based on the pedigree-based and marker-based information are depicted in the heat maps in Figure 2. The values of the pedigree-based relationship matrix are derived from the probability of identity by descent (IBD) and therefore the values are between zero and one. Values of zero mean no relationship and one indicate an identical relationship. Unlike the pedigree-based matrix, values of the marker matrix are composed of negative and positive values. The negative relationships are explained from the centering of the marker covariates, leading to centering of the entire marker-based matrix such that the sum of all elements in the matrix is zero. Negative values in the marker-based relationship matrix mean that detection of an allele in one line makes it less likely to be detected in the other line, zero means a lack of dependence and positive values indicate an increased likelihood of the allele being detected in the other line.

The pedigree heat map shows fewer groupings due to the assumptions of IBD that allow full siblings derived from the same cross to be similar. The marker heat map, however, shows many more groupings. This was to be expected because the marker matrix accounts for Mendelian segregation of alleles, such that full siblings that are more or less expected due to random sampling can be distinguished. The heat maps of both matrices showed a closer relationship between NS lines (Figure 2A) than SJ lines (Figure 2B), as is evident from the greater density of blue and yellow shades in the NS heat maps compared to SJ. Moreover, the mass of SJ lines at zero (lack of dependence), as shown in the color key density plot for the marker matrix, were higher than that of NS, indicating a higher relatedness of NS lines compared to SJ lines.

### Variance Component Estimates

A summary of the variance components for the studied traits estimated from all six models using combined data and individual company data are presented in Tables 3A–C, respectively. The main effects of environments consistently explained most of the total variance for all traits. Using SJ data alone, environmental variance (*E*) explained between 42% (in SF_abv2.2 *ELGA-GxE-AxE* model) and 81% (in protein *ELG* model) approximately of the total variance (Table 3B). Environmental variance explained between 36% (in SF_abv2.2 for *ELA-AxE* model) and 63% (in protein *ELG* model) approximately of the total variance using NS data (Table 3C). Due to the large proportions of variance explained by the environments in the different models, the proportional contribution of each of the random effects relative to the total phenotypic (within-environment) variance was estimated, as suggested in literature (Jarquín et al., 2014; Pérez-Rodríguez et al., 2015).

**TABLE 3A:** Variance components and percentage within-environment variance for random effects in the models with combined data.

Trait	Model	Estimated variance components							Percentage of within-environment variance^*r*^					
			
		*E*	*L*	*G*	*GxE*	*A*	*AxE*	*R*	*L*	*G*	*GxE*	*A*	*AxE*	*R*
Protein	ELG	0.59	0.03	0.06				0.12	14.82	28.49				56.69
	ELG-GxE	0.60	0.03	0.06	0.02			0.11	13.59	26.99	7.92			51.50
	ELA	0.58	0.03			0.08		0.12	12.91			35.35		51.74
	ELA-AxE	0.56	0.03			0.08	0.02	0.11	11.35			34.61	6.99	47.04
	ELGA	0.58	0.02	0.04		0.03		0.12	9.25	20.26		16.12		54.37
	ELGA-GxE-AxE	0.58	0.02	0.04	0.01	0.03	0.01	0.10	8.07	18.97	5.89	14.19	5.57	47.31
Test weight	ELG	10.10	0.68	2.00				2.51	13.14	38.52				48.34
	ELG-GxE	10.30	0.62	1.96	0.35			2.32	11.82	37.36	6.60			44.22
	ELA	9.93	0.94			2.12		2.54	16.71			37.89		45.40
	ELA-AxE	10.10	0.88			2.11	0.29	2.41	15.43			37.09	5.12	42.36
	ELGA	9.77	0.47	1.51		0.73		2.46	9.03	29.20		14.19		47.57
	ELGA-GxE-AxE	9.74	0.40	1.53	0.27	0.67	0.23	2.22	7.47	28.79	5.04	12.68	4.23	41.78
SF_abv2.5	ELG	96.00	16.30	31.70				28.10	21.42	41.66				36.93
	ELG-GxE	90.20	15.70	29.90	4.11			25.70	20.82	39.65	5.45			34.08
	ELA	90.60	12.40			50.90		28.10	13.57			55.69		30.74
	ELA-AxE	88.20	11.30			50.80	3.64	26.30	12.28			55.19	3.95	28.57
	ELGA	88.10	8.52	22.50		17.30		27.70	11.21	29.60		22.76		36.44
	ELGA-GxE-AxE	83.80	7.62	22.10	3.17	16.20	2.90	24.60	9.95	28.85	4.14	21.15	3.79	32.12
SF_abv2.2	ELG	3.97	1.31	1.43				2.32	25.89	28.26				45.85
	ELG-GxE	3.85	1.27	1.35	0.26			2.16	25.19	26.78	5.20			42.84
	ELA	3.85	0.87			2.82		2.30	14.52			47.08		38.40
	ELA-AxE	3.60	0.78			2.75	0.26	2.17	13.12			46.14	4.33	36.41
	ELGA	3.67	0.68	1.01		1.28		2.29	12.91	19.21		24.34		43.54
	ELGA-GxE-AxE	3.52	0.61	0.97	0.20	1.17	0.21	2.07	11.65	18.61	3.82	22.38	3.96	39.59

**E* environment, *L* line, *G* genomic (marker), *A* pedigree information, *GxE* genomic by environment interaction, *AxE* pedigree × environment interaction, and *R* model residual. ^*r*^Relative to the total variance minus the variance due to environment main effect.*

**TABLE 3B:** Variance components and percentage within-environment variance for random effects in the models with SJ data.

Trait	Model	Estimated variance components							Percentage of within-environment variance^*r*^					
			
		*E*	*L*	*G*	*GxE*	*A*	*AxE*	*R*	*L*	*G*	*GxE*	*A*	*AxE*	*R*
Protein	ELG	1.02	0.05	0.06				0.15	17.78	21.88				60.33
	ELG-GxE	1.00	0.04	0.05	0.03			0.14	15.14	19.37	11.30			54.19
	ELA	0.96	0.05			0.07		0.16	18.24			25.90		55.87
	ELA-AxE	0.94	0.05			0.07	0.03	0.15	15.99			22.72	10.30	50.99
	ELGA	0.95	0.04	0.04		0.05		0.15	13.12	15.20		16.51		55.17
	ELGA-GxE-AxE	0.94	0.03	0.04	0.02	0.04	0.02	0.13	10.32	12.96	8.18	13.62	7.87	47.05
SSW	ELG	11.40	0.93	1.40				4.17	14.32	21.54				64.14
	ELG-GxE	10.60	0.82	1.33	0.51			4.06	12.21	19.80	7.56			60.43
	ELA	11.10	1.05			1.76		4.32	14.73			24.68		60.59
	ELA-AxE	10.20	0.97			1.64	0.49	4.24	13.21			22.34	6.70	57.75
	ELGA	10.50	0.72	1.01		1.00		4.10	10.60	14.78		14.62		60.00
	ELGA-GxE-AxE	10.90	0.62	0.93	0.41	0.92	0.38	3.95	8.58	12.89	5.68	12.78	5.31	54.75
SF_abv2.5	ELG	118.00	15.70	28.20				37.40	19.31	34.69				46.00
	ELG-GxE	108.00	13.80	26.80	6.88			34.60	16.81	32.65	8.38			42.15
	ELA	116.00	14.60			42.00		37.70	15.48			44.54		39.98
	ELA-AxE	95.40	12.80			39.40	7.13	34.80	13.60			41.86	7.57	36.97
	ELGA	121.00	9.80	17.80		19.20		36.20	11.81	21.45		23.13		43.61
	ELGA-GxE-AxE	92.70	7.93	16.40	5.12	16.80	5.56	32.10	9.45	19.54	6.10	20.02	6.63	38.26
SF_abv2.2	ELG	5.04	0.94	1.40				3.24	16.88	25.08				58.04
	ELG-GxE	4.46	0.78	1.37	0.45			3.06	13.82	24.18	7.98			54.02
	ELA	5.11	0.85			2.15		3.23	13.62			34.52		51.86
	ELA-AxE	4.48	0.73			1.89	0.49	3.04	11.79			30.75	8.00	49.46
	ELGA	4.62	0.61	0.83		1.19		3.14	10.51	14.39		20.64		54.46
	ELGA-GxE-AxE	4.10	0.47	0.78	0.32	0.95	0.39	2.88	8.05	13.44	5.51	16.38	6.75	49.87

**E* environment, *L* line, *G* genomic (marker), *A* pedigree information, *GxE* genomic by environment interaction, *AxE* pedigree × environment interaction, and *R* model residual. ^*r*^Relative to the total variance minus the variance due to environment main effect.*

**TABLE 3C:** Variance components and percentage within-environment variance for random effects in the models with NS data.

Trait	Model	Estimated variance components							Percentage of within-environment variance^*r*^					
			
		*E*	*L*	*G*	*GxE*	*A*	*AxE*	*R*	*L*	*G*	*GxE*	*A*	*AxE*	*R*
Protein	ELG	0.31	0.03	0.05				0.10	16.41	27.06				56.53
	ELG-GxE	0.29	0.03	0.05	0.02			0.09	15.63	26.12	8.31			49.95
	ELA	0.32	0.03			0.07		0.10	13.17			36.13		50.70
	ELA-AxE	0.33	0.02			0.07	0.02	0.09	11.98			35.98	7.66	44.38
	ELGA	0.29	0.02	0.03		0.03		0.10	10.55	17.34		17.72		54.39
	ELGA-GxE-AxE	0.29	0.02	0.03	0.01	0.03	0.01	0.09	9.14	15.37	6.13	18.12	6.23	45.01
SSW	ELG	4.83	0.62	2.00				1.62	14.70	47.13				38.17
	ELG-GxE	4.86	0.57	1.98	0.30			1.43	13.29	46.34	6.90			33.47
	ELA	4.84	0.86			2.35		1.64	17.73			48.45		33.81
	ELA-AxE	4.83	0.81			2.37	0.27	1.46	16.40			48.29	5.56	29.75
	ELGA	4.48	0.42	1.59		0.65		1.61	9.93	37.25		15.11		37.71
	ELGA-GxE-AxE	4.41	0.37	1.65	0.22	0.57	0.22	1.34	8.47	37.78	5.13	12.96	4.97	30.68
SF_abv2.5	ELG	84.50	14.50	30.30				24.40	20.95	43.79				35.26
	ELG-GxE	79.60	14.40	30.00	4.09			21.90	20.46	42.62	5.81			31.11
	ELA	89.30	14.30			48.20		24.40	16.46			55.47		28.08
	ELA-AxE	82.90	13.10			50.30	3.81	22.40	14.62			56.13	4.25	25.00
	ELGA	80.40	9.64	24.00		13.50		24.20	13.51	33.64		18.92		33.92
	ELGA-GxE-AxE	73.50	9.24	24.70	3.29	12.80	2.96	21.00	12.49	33.38	4.45	17.30	4.00	28.38
SF_abv2.2	ELG	3.54	1.30	1.37				1.93	28.26	29.78				41.96
	ELG-GxE	3.15	1.31	1.35	0.27			1.75	27.99	28.84	5.79			37.39
	ELA	3.86	0.98			2.85		1.93	17.00			49.49		33.51
	ELA-AxE	3.25	0.93			2.86	0.26	1.76	15.93			49.26	4.50	30.31
	ELGA	3.10	0.86	1.13		1.02		1.92	17.36	22.94		20.71		38.98
	ELGA-GxE-AxE	2.89	0.86	1.10	0.22	0.92	0.20	1.67	13.89	22.18	4.34	18.47	4.03	33.68

**E* environment, *L* line, *G* genomic (marker), *A* pedigree information, *GxE* genomic by environment interaction, *AxE* pedigree × environment interaction, and *R* model residual. ^*r*^Relative to the total variance minus the variance due to environment main effect.*

Depending on the model and trait, lines explained between 8% and 19% (with SJ data), and between 8.5 and 28% (with NS data) of the within-environment variance. Marker information explained between 12.9 and 34.7% of the within-environment variance, while pedigree information explained between 12.8 and 44.5% (for SJ data), and depending on the trait and the inclusion (or not) of interaction effects. With NS data on the other hand, marker information explained between 15.4 and 43.8% while pedigree information explained between 13 and 49.5% of the within-environment variance.

There was a decrease in the estimated within-environment error variance (*R*) when interaction terms were included in the main effect models for all traits. Considering protein in the SJ data, the inclusion of the interaction terms *GxE* and *AxE* to models *ELG* and *ELA* induced a reduction of approximately 10% and 8.7% in the estimated residual variance, respectively. The greatest reduction in residual variance (14.7%) due to interaction effects was observed in the most comprehensive model. A similar trend was observed for all the other traits in SJ and NS data as well. However, the percentage of total variance explained by interaction effect in SJ was slightly higher than that of NS. This indicated that not all the phenotypic variation could be captured fully by main effects alone. A substantial amount of error variance reduction related to the *ELG*, *ELA*, and *ELGA* models could be attributed to genetic-environment interaction, hence the need to factor them into the study.

In the models that included both marker and pedigree effects, the total additive genetic variance was partitioned into fractions due to marker and pedigree information (Table 4). Both main effect models and interaction effect models were analyzed. The proportion of the additive variance due to the markers and pedigree information was estimated as the ratio of marker (genomic) or pedigree variance to the total additive variance, respectively. In the combined data, most of the total additive genetic variance (average of 60% for the three traits) was attributed to marker effects.

**TABLE 4:** Partition of total additive genetic variance.

Traits	Model ELGA				Model ELGA-GxE-AxE			
		
	$\sigma_{G}^{2}$	$\sigma_{A}^{2}$	*VR*_*G*_	*VR*_*A*_	$\sigma_{G}^{2}$	$\sigma_{A}^{2}$	*VR*_*G*_	*VR*_*A*_
**Combined data**								
Protein	0.044	0.035	0.557	0.443	0.041	0.031	0.572	0.428
Test weight	1.510	0.734	0.673	0.327	1.530	0.674	0.694	0.306
SF_abv2.5	22.500	17.300	0.565	0.435	22.100	16.200	0.577	0.423
SF_abv2.2	1.010	1.280	0.441	0.559	0.973	1.170	0.454	0.546
**SJ**								
Protein	0.042	0.045	0.479	0.521	0.037	0.039	0.487	0.513
Test weight	1.010	0.999	0.503	0.497	0.930	0.922	0.502	0.498
SF_abv2.5	17.800	19.200	0.481	0.519	16.400	16.800	0.494	0.506
SF_abv2.2	0.830	1.190	0.411	0.589	0.776	0.946	0.451	0.549
**NS**								
Protein	0.032	0.033	0.495	0.505	0.029	0.034	0.459	0.541
Test weight	1.590	0.645	0.711	0.289	1.650	0.566	0.745	0.255
SF_abv2.5	24.000	13.500	0.640	0.360	24.700	12.800	0.659	0.341
SF_abv2.2	1.130	1.020	0.526	0.474	1.100	0.916	0.546	0.454

*$\sigma_{G}^{2}$: additive variance traced by marker(genomic) matrix, *$\sigma_{A}^{2}$* additive variance traced by pedigree matrix. *VR*: ratio of additive genetic (genomic or pedigree) variance in model over total additive genetic variance.*

However, a different scenario was observed when data from the different breeding companies were analyzed separately. Averaged across all four traits in the SJ data, 53% of the total additive genetic variance in the main effect model was attributed to the pedigree relationships among the lines. In NS data, however, more than half of the total additive genetic variance for all the traits, with the exception of protein, in the main effect model was attributed to the genomic/marker relationships among the lines. The scenarios observed with the interaction models were similar to the main effect models. This indicated that both the genomic and pedigree relationship matrices were able to capture significant proportions of the additive genetic variance in the population with the relative importance of genomic and pedigree information varying by trait and breeding company used in the current study.

### Heritability

Broad-sense and narrow-sense heritabilities were both estimated from the variance component analysis, with the former based on the sum of the line variance and the pedigree and/or marker variance. Whereas marker and pedigree capture the true additive genetic effect, the lines capture the genetic residual effect (Oakey et al., 2016; Hunt et al., 2018). Broad-sense heritability is needed to adjust prediction ability when computing prediction accuracy, and they can be inferred from the variance components estimates (data not shown).

**FIGURE 3:**
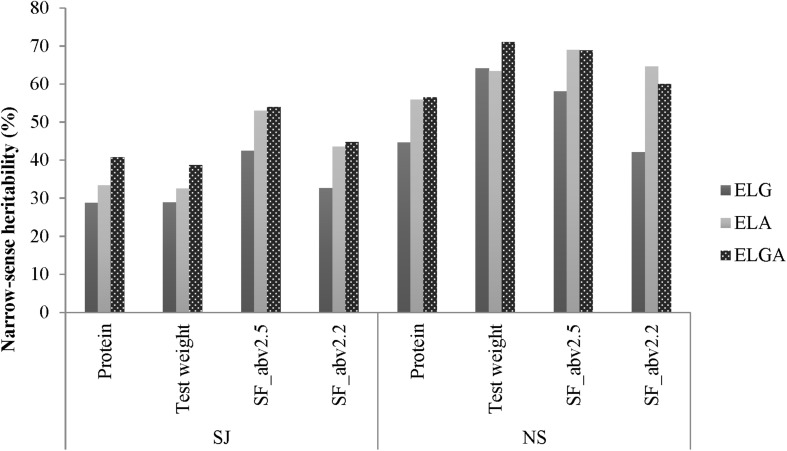
Narrow-sense heritability estimates for all traits estimated with SJ and NS.

Figure 3 shows the narrow-sense heritability *h*^2^ based on individual line records estimated from the main effect models for all traits for the separate breeding programs. The models compared were those that used marker relationships alone, pedigree relationships alone, and combined marker and pedigree relationships for all the traits.

Overall, *h*^2^ in NS was higher than in SJ for all the traits measured. In SJ, *h*^2^ was highest for SF_abv2.5, followed by SF_abv2.2, test weight, and protein. In NS, *h*^2^ was highest for test weight, followed by SF_abv2.5, protein, and SF_abv2.2. As expected, *h*^2^ traced by pedigree information was higher than that traced by marker information for all the traits in the SJ data. Overall, *h*^2^ was reduced by almost 29, 19, 20, and 25% for protein, test weight, SF_abv2.5, and SF_abv2.2, respectively, when the marker relationship matrix *ELG* was employed rather than the pedigree relationship matrix (*ELA*). It was also observed that *h*^2^ traced by combined pedigree and marker information was higher than using either of the matrices on their own.

In NS, however, except for test weight *h*^2^ traced by pedigree information alone was higher than marker information alone for protein, SF_abv2.5, and SF_abv2.2. In addition, combining pedigree and marker information improved *h*^2^ compared to marker or pedigree information alone for protein and test weight; however, in the seed fractions there was no gain compared to the pedigree information.

### Within-Company Predictions Using Different Cross Validations

**FIGURE 4:**
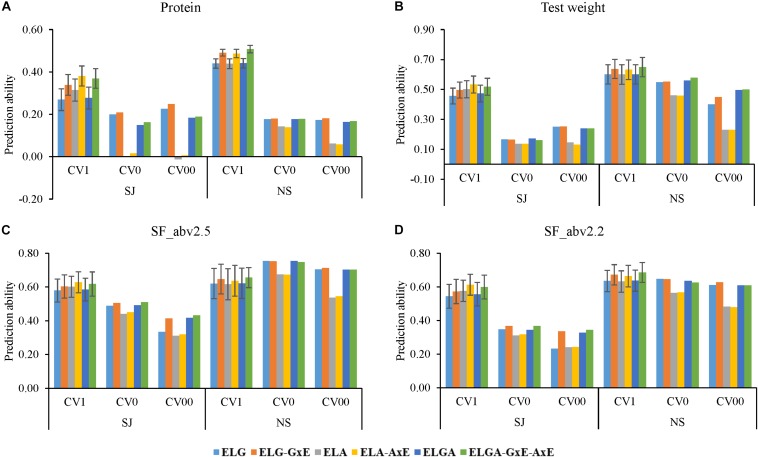
Prediction ability obtained using six models for the three cross-validation schemes in the two breeding programs for all traits. CV1; predicting newly developed lines in known environments, CV0; predicting unknown (future) year, and CV00; predicting newly developed lines in unknown future year prediction abilities for **(A)** Protein, **(B)** Test weight, **(C)** Seed fraction SF_abv2.5, and **(D)** Seed fraction SF_abv2.2. Error bars depict the standard error from the mean of the 10 fold correlations.

Prediction results for the different cross validation schemes from the six models using data from the two breeding programs (NS and SJ) separately (within-company predictions) for all traits are presented in Figure 4.

Generally, predictions in all the cross-validation schemes for NS were better than in SJ. For instance, considering protein, the average prediction ability across models were 0.32, 0.12, and 0.14 for CV1, CV0, and CV00, respectively, for SJ compared to 0.47, 0.17, and 0.13 for CV1, CV0, and CV00, respectively, in NS. In test weight, the average predictive abilities across models were 0.50 (CV1), 0.16 (CV0), and 0.21 (CV00) for SJ but 0.62 (CV1), 0.53 (CV0), and 0.38 (CV00). In SF-abv2.5, predictive ability across models in SJ were 0.60. 0.48, 0.37 for CV1, CV0, and CV00, respectively. Similarly, in SF_abv2.2, the average predictive ability across models were 0.63, 0.73, and 0.65 for CV1, CV0, and CV00, respectively, in NS.

### Prediction by Cross-Validation Scheme CV1

In predicting newly developed lines in CV1, pedigree information alone models (*ELA*) performed better (SJ data) or similar (NS data) to marker information alone models (*ELG*) for all traits. In SJ, prediction ability for *ELG* in CV1 were 0.27, 0.46, 0.58, and 0.54 for protein, test weight, SF_abv2.5, and SF_abv2.2, respectively, while prediction ability for *ELA* in CV1 were 0.31, 0.50, 0.60, and 0.58 for protein, test weight, SF_abv2.5, and SF_abv2.2, respectively, (Figure 4). In NS on the other hand prediction ability for *ELG* in CV1 were 0.44, 0.60, 0.62, and 0.64 for protein, test weight, SF_abv2.5, and SF_abv2.2, respectively, while prediction ability for *ELA* in CV1 was similar to *ELG* for all the traits. Combining pedigree and marker information (*ELGA*) improved prediction ability compared with models using marker information alone, but not compared with models using pedigree information alone for SJ data for all traits. In NS, *ELGA* models performed similarly to *ELG* and *ELA* models for all traits.

Inclusion of interaction effects increased predictions compared to the main effect models for all traits in the CV1 scheme for both SJ and NS. The greater prediction performance from the inclusion of interaction effect with the main effect models was found to be highest for protein in in both SJ and NS. In addition, the gain in prediction performance due to the addition of interaction was better in SJ than NS for most of the traits. For instance, in SJ, *GxE* improved the prediction ability of the *ELG* model by approximately 26, 9, 4, and 5% for protein, test weight, SF_abv2.5 and SF_abv2.2, respectively, while *AxE* improved the ability of the *ELA* model by 21, 6, 4, and 6% for protein, test weight, SF_abv2.5, and SF_abv2.2, respectively. In NS, inclusion of *GxE* in the *ELG* model increased prediction ability by 12, 6, 4, and 6%, while AxE increased the prediction ability of the *ELA* model by 11, 5, 3, and 5% for protein, test weight, SF_abv2.5, and SF_abv2.2, respectively. Thus, the models that captured the interactions performed better than their corresponding main effect models for all traits.

### Prediction by Cross-Validation Schemes CV0 and CV00

The scheme for predicting performance of lines in future year (CV0) and the scheme for predicting the performance of new lines in future year (CV00) showed a different pattern compared to CVI. For all traits in both SJ and NS, marker alone models outperformed pedigree alone models. The average predictive ability for protein content in both companies was 0.19 and 0.21 for marker models in CV0 and CV00, respectively, while with pedigree models; it was 0.07 and 0.03 for CV0 and CV00, respectively. In test weight, the average predictive ability for marker models in both breeding programs was 0.36 (CV0) and 0.34 (CV00) while the average predictive ability for pedigree models was 0.30 (CV0) and 0.18 (CV00). The trend was similar for the other traits.

In NS, though *ELGA* models performed better than *ELA* models, there was no advantage in predictive ability compared to *ELG* models for all the traits under CV0 and CV00 schemes. In SJ on the other hand, *ELGA* always performed better than *ELA* but its performance against *ELG* was inconsistent depending on the trait. In protein, ELGA performed worse than *ELG* while in test weight, *ELGA* performed similar to *ELG* for both CV0 and CV00. In SF_abv2.5 and SF_abv2.2, *ELGA* performed similar to *ELG* in CV0 but better in CV00.

Interestingly in CV0 and CV00, inclusion of interaction effects did not always improve prediction performance as seen in the case of CV1 (Table 5). In most cases, the addition of interaction effect in CV0 and CV00 caused a decrease in prediction performance. In cases where there was an increase due to interaction, the gains were always lower compared to CV1 with a few exceptions in SF_abv2.5 and SF_abv2.2. For instance, in SJ the percentage change in predictive ability were between approximately -158% (CV00 *AxE*) to 10% (CV00 *GxE*) while that in CV1 ranged between approximately 21% (*AxE*) to 33% (*GxE-AxE*). In NS, the percentage change in predictive ability due to interaction ranged between approximately 11% (*AxE*) to 15% (*GxE-AxE*) for protein content in CV1 and between -6% (CV00 *AxE*) to 5% (CV00 *GxE*) for the forward predictions.

**TABLE 5:** Percentage change in prediction ability of main effect models with the inclusion of interaction effects.

		SJ			NS		
			
		GxE	AxE	GxE-AxE	GxE	AxE	GxE-AxE
Protein	CV1	26.02	21.34	33.21	11.59	10.93	15.19
	CV0	5.03	0.00	8.72	1.13	−2.11	0.56
	CV00	10.18	−158.33	2.72	4.62	−6.15	2.44
Test weight	CV1	8.77	6.39	9.51	5.99	5.33	7.99
	CV0	−0.60	0.00	−6.94	0.73	−0.43	3.58
	CV00	0.80	−10.34	−0.42	12.25	0.00	0.60
SF_abv2.5	CV1	4.15	4.32	5.64	4.35	3.41	5.47
	CV0	3.27	2.27	3.87	−0.13	−0.15	−0.80
	CV00	23.95	2.57	3.60	1.28	1.49	0.00
SF_abv2.2	CV1	5.33	6.42	7.73	5.83	5.06	7.69
	CV0	5.76	2.25	6.98	−0.15	0.53	−1.42
	CV00	44.21	1.25	4.88	2.62	−0.83	0.00

### Comparing Within-Company and Across-Company Predictions Using CV1

The effect of combining populations to increase training set size in prediction was assessed by comparing predictive ability estimated using within-company (lines from either one breeding program) or across-company (lines from both breeding programs). Training sets that included lines from combined breeding programs increased the entries in that training set by almost twice compared to training sets that included lines from a single breeding company. The validation sets always contained lines from either of the breeding programs. The hypothesis here was that training sets from combined populations would increase prediction performance compared to training sets from single populations since by combining populations, the size of the training set is increased.

**FIGURE 5:**
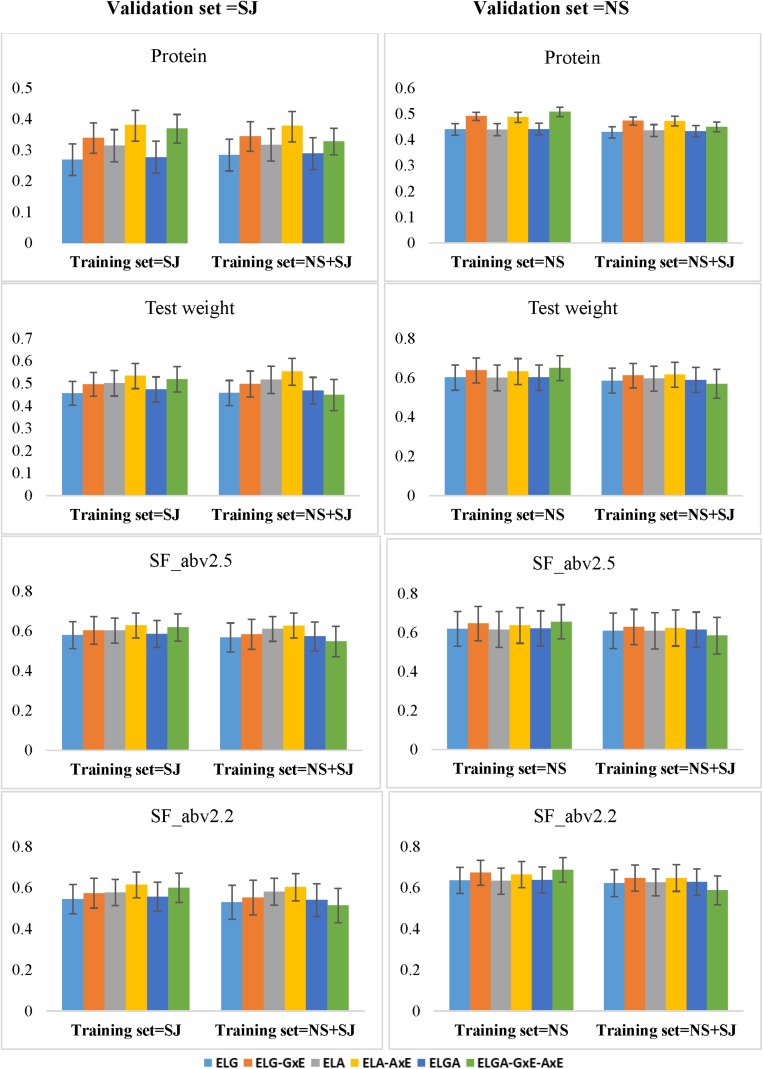
Prediction abilities (*y*-axis) obtained when the training set is composed of lines from either one breeding program or combined breeding programs. Using the CV1 scheme (predicting newly developed lines in known environments), all six models were compared for all the traits. The *x*-axis displays different training sets. For the left panels (Validation set = SJ), *N* = 392 for SJ training set and *N* = 826 for NS + SJ training set. For the right panels (Validation set = NS), *N* = 438 for NS training set, and *N* = 826 for NS + SJ training set. Error bars depict the standard error from the mean of the 10 fold correlations.

Our results were mostly population and trait specific. When the validation is from SJ, then combining populations from NS, and SJ either did not change or slightly increased prediction ability in most of the models (except for the most comprehensive model) for protein and test weight. In SF_abv2.5 and SF_abv2.2, increasing training set size by combining populations from SJ and NS reduced prediction ability in most of models (Figure 5). When the validation set is from NS, increasing training set size by combining SJ and NS reduced prediction ability in all the traits for all the models. In the most comprehensive model *ELGA-GxA-AxE*, there was a consistent reduction in prediction ability by combining populations from both breeding programs to predict performance of lines from either of the programs. With the exception of *ELGA-GxA-AxE*, performance of the other models was similar to the results obtained from the within-company prediction.

## Discussion

### Variance Components and Heritability

This study explored several models to determine the effect of independent and combined marker and pedigree relationships on prediction, as well as the effect of environmental interaction terms. From the results of the variance component analysis, the inclusion of the interaction of markers and environments or pedigree and environments was found to induce a remarkable reduction in unexplained variance for all traits. A similar decline has also been seen in previous studies in wheat (Burgueño et al., 2012; Jarquín et al., 2014; Sukumaran et al., 2017) and cotton (Pérez-Rodríguez et al., 2015). It was also observed that interaction terms contributed to the total within-environment phenotypic variance to differing degrees. This indicated that models with only main effects were not adequate to capture most of the variance in the model. Hence, the introduction of interaction terms meant that an increased proportion of the model variance could be captured, which is likely to increase prediction ability.

Models using both marker and pedigree information increased heritability for most of the traits compared to models with either marker or pedigree information alone. Similar results were also observed in sorghum (Velazco et al., 2019) where a *k* matrix composed of combined pedigree and marker information increased trait heritabilities. Increased heritability due to combined marker and pedigree relationships indicated that such models have a tendency to capture greater genetic variation than using either marker or pedigree information alone.

The reduction in overall narrow-sense heritabilities when *ELG* was employed compared with *ELA* in this study reflects the notion of a pedigree-based relationship matrix not adjusting for Mendelian sampling, thus leading to a likely overestimation of additive genetic variance. The general consensus in the literature is that marker-derived relationship information is evolving to becoming preferred in genomic prediction research to pedigree-derived relationship information (Crossa et al., 2010; Albrecht et al., 2011; Burgueño et al., 2012; Auinger et al., 2016; Velazco et al., 2019). This is because, unlike pedigree-derived relationship information, marker-derived relationship information accounts for common ancestry, which is otherwise not considered in the pedigree. Moreover, the marker-derived matrix particularly accounts for departures of realized genetic relationships from their expected values due to Mendelian segregation (Burgueño et al., 2012; Velazco et al., 2019), and thus providing relatively accurate genetic variance components compared with the pedigree-derived matrix (El-Dien et al., 2016).

### Model Performance Under Different Cross-Validation Schemes

The trend for model performance in terms of prediction ability was similar for all the traits with the different schemes exhibiting differences in how the models performed. Results from this study showed that model performance was dependent on the cross-validation scheme used. In general, prediction of NS lines was better than SJ lines for most of the traits. This was driven by the close relationship observed between lines in the matrices for NS, as shown in Figure 2. With a low number of subfamilies within NS compared to SJ, the number of full siblings within each subfamily unit in NS was relatively high compared to SJ, thus contributing to lines from NS being predicted more accurately than SJ. This was to be expected because NS has a more recent barley-breeding nursey with narrower genetic base comprised mostly of Northern Europe varieties, while SJ barley breeding has a much longer history with a broader genetic base consisting of genotypes originating from all over Europe.

In CV1, pedigree information was superior or competitive with marker information. In SJ data, pedigree models performed better than marker models while in NS, pedigree models performed similarly to marker models. This result is consistent with previous observations in wheat and sorghum (Juliana et al., 2017; Hunt et al., 2018; Howard et al., 2019). CV0 and CV00 involve more complex prediction situations that span across different environments (years), and in these situations marker information models performed better than pedigree information models. In these complex situations, the number of related lines (full and half siblings) in the training set reduces when sampling across years, and hence a clear advantage of the realized relationship captured by marker is seen (Juliana et al., 2018). When exceptional pedigree records are kept that go back several generations, pedigree-based models may perform better than marker-based models (Juliana et al., 2017). However, this will only hold if the number of related lines in the training and validation sets is optimal. Thus, when lines are derived from a pedigree that contains diverse siblings, they are more likely to be predicted accurately using marker information than using pedigree information, in which case all full siblings within a single subfamily would have the same predictions (Hunt et al., 2018).

Dense marker coverage is a prerequisite for improved prediction accuracies (Heffner et al., 2009). However, the amount of additive genetic variance that can be explained by the markers not only depends on dense marker coverage, but also on the number of markers in linkage disequilibrium (LD) with causative genes (Jensen et al., 2012). This implies that when markers in LD with causative genes are low, models based solely on markers may perform worse than their corresponding models based only on pedigree. Although it was assumed that the number of markers used in this study provided excellent genome-wide coverage, there is a possibility that these markers insufficiently covered some major regions associated with the traits, thus leading to lower prediction accuracies in some of the models based only on markers. This was evident by most of the total additive genetic variance being explained by pedigree relationships and not marker relationships (Table 4) when the models included both matrices especially in the SJ data.

Combining marker and pedigree information did not show any advantage in prediction ability. Several studies have reported the improvement of prediction by a combination of marker and pedigree relationships with respect to models with marker or pedigree information alone (Crossa et al., 2010; Albrecht et al., 2011; Burgueño et al., 2012; Auinger et al., 2016; Juliana et al., 2017; Velazco et al., 2019). The reported improvement in prediction of combined marker and pedigree information models is based on the assumption that if markers are not in full LD with QTLs, the addition of pedigree relationship information will contribute to capturing associations between causative alleles because of common ancestry identity, which can improve prediction accuracies (Velazco et al., 2019). However, in the presence of high-quality genomic markers and pedigree information, there is a possibility of some redundancy between the regression on markers and that of pedigree, leading to no or only a marginal gain (Albrecht et al., 2011; Juliana et al., 2017). The lack of advantage when both matrices were combined in this study could be attributed to redundancy in the information captured by both (Crossa et al., 2017) or due to issues of collinearity arising from them since they are not mutually orthogonal (Howard et al., 2019).

Assessing and quantifying genotype-by-environment interaction is an integral part of GS and has been shown to lead to increase in prediction ability (Jarquín et al., 2017). In this study, inclusion of genotype-by-environment interaction terms to the main effect models led to increase in prediction abilities for all the traits in CV1. Protein, which had the most dramatic reduction in within-environment error variance following the addition of interaction terms, had the most dramatic gain from interaction terms with respect to prediction ability in both NS and SJ. This gain in prediction ability was not surprising, given the contribution of the interaction terms to the main effect models in the variance component analysis, as discussed previously. Moreover, in CV1, the most comprehensive model (*ELGA-GxE-AxE*) had the highest prediction ability across all traits. Bearing in mind the extent of the *GxE* and *AxE* variance contribution in each of the traits, this increase was not a surprise. Thus, it appears that if combining marker and pedigree is favorable, sizable gains in prediction ability can be attained by jointly considering their environmental interactions in barley, as has also been observed in previous studies in wheat (Burgueño et al., 2012; Sukumaran et al., 2017).

Interestingly, accounting for genotype-by-environment interaction did not always improve prediction ability in CV0 and CV00. Similar results have been reported in wheat (Howard et al., 2019) and this could be because CV1 which involves the prediction of newly developed lines in tested environments allows borrowing of information from other environments (years) while CV0 and CV00 which involves the prediction of performance of unobserved environments, a much more complex situation does not.

### Increasing Training Set Size by Combining Populations

Several studies have shown that the increasing training set size improves prediction ability (Zhong et al., 2009; Asoro et al., 2011; Zhang et al., 2017). Due to this notion, it was hypothesized that increasing the training set size by combining lines from the different breeding programs would improve prediction ability.

The results indicated that increasing training set size by combining different populations produced no benefit in terms of predicting each population test set. In most cases, it led to either a no change in prediction ability or a decrease. The loss in prediction ability of across-company compared to within-company ranged from 0.7 to 4.6% on average when the validation set comprised of SJ data and from 3.5 to 4.7% when the validation set comprised NS data. This finding is similar to what has been observed in GS for Fusarium head blight resistance in six-rowed barley (Lorenz et al., 2012) and GS for grain yield in bread wheat (Juliana et al., 2018). Conclusions from both studies suggest that the lack of advantage in combining populations corresponding to different breeding programs to increase the training population could be due to the possibility of marker effects differing across the populations and hence causing substantial QTL-by-environment interaction. Other reasons could be due to different QTLs segregating between the two populations and different QTL effects between the different populations due to epistasis (Lorenz et al., 2012).

In contrast to these results, others have shown that prediction ability can be increased by combining multiple populations. In animal breeding, genomic selection has been shown to benefit from combining populations when the genetic distance between the breeds is close. In distance related breeds, genomic prediction can be improved if the models used are those that put more focus on genomic markers in strong LD with causative variants (Lund et al., 2014). Similarly, the advantage from combining populations can surge with increasing number of markers, increasing heritability and decreasing divergence time between the populations. With an increased marker density, even very diverged populations are likely to share marker-QTL LD structure that was generated due to mutational events that occurred before the divergence of the population (de Roos et al., 2009). This in effect reduces the negative influence of contrasting QTL-marker effects between the populations and subsequently reduces recombination frequency between markers and QTLs.

The populations used in this study were assumed to be quite related since they come from competing breeding companies in close proximity targeting the agro-climatic zones of Northern Europe. There is high probability of both companies sharing common lines as founders since they have the same consumer target and the possibility of varieties released by one company being used as a source of new genetic material for crosses in the population of the other (Robertsen et al., 2019). However, from our results we can deduce that the populations are not related enough to benefit from this combination. By exploring more sophisticated variable selection methods, the advantage of increasing training population size by combining lines from both breeding programs can be realized, since such models are able to capture tightly linked QTL-marker effects.

## Data Availability Statement

The datasets generated for this study can be found in the article/Supplementary Material.

## Author Contributions

TA-Y, SR, and LJ conceptualized the idea for the manuscript. TA-Y analyzed the data and wrote the manuscript. LJ supported in the statistical analysis. RH and JJ designed the field trials and provided genomic and pedigree data. RH, JJ, and TA-Y collected the phenotypic data. All authors read and approved the final manuscript.

## Conflict of Interest

JJ was employed by the company Nordic Seed A/S and author RH was employed by the company Sejet Plant Breeding A/S. The remaining authors declare that the research was conducted in the absence of any commercial or financial relationships that could be construed as a potential conflict of interest.

## Supplementary Material

Material S1 Phenotypic data, genotypic data and relationship matrices based on pedigree information from the two breeding companies.
